# Effect of cigarette smoking on subgingival bacteria in healthy subjects and patients with chronic periodontitis

**DOI:** 10.1186/s12903-017-0359-4

**Published:** 2017-03-21

**Authors:** Jumana A. Karasneh, Rola A. Al Habashneh, Nour Aldain S. Marzouka, Martin H. Thornhill

**Affiliations:** 1 Department of Oral Medicine & Oral Surgery, Faculty of Dentistry, Jordan University of Science & Technology, Irbid, Jordan; 2 Department of Preventive Dentistry, Faculty of Dentistry, Jordan University of Science and Technology, Irbid, Jordan; 3 Department of Medical Sciences, Uppsala University, Uppsala, Sweden; 4 Unit of Oral and Maxillofacial Medicine and Surgery, The University of Sheffield, School of Clinical Dentistry, Sheffield, UK

**Keywords:** Bacteria, Cigarette smoking, Chronic periodontitis, Polymerase chain reaction, 16S ribosomal RNA

## Abstract

**Background:**

Cigarette smoking is known to increase the risk of periodontal destruction and developing chronic periodontitis (CP). It is also reported to affect the subgingival bacterial profile among CP patients. However, studies on the effect of smoking on the bacterial profile among healthy subjects are still limited. Therefore, the aim of this study was to investigate the impact of smoking on the subgingival bacterial profile in both healthy adults and CP patients.

**Methods:**

Subgingival plaque samples were collected from CP patients (30 nonsmokers and 9 smokers) and healthy subjects (37 non-smokers and 18 smokers). Genomic DNA was extracted and 25 bacterial species were detected using PCR of 16S rRNA. Comparing smokers to non-smokers from each group was conducted using chi2 and binary logistic regression analysis.

**Results:**

After correcting for confounding factors, the odds of having *Slackia exigua*, *Selenomonas sputigena* and *Campylobacter rectus* was higher among healthy smokers (ORadj = 10.1, 6.62 and 5.62 respectively). While for CP group, the highest odds were observed for *Treponema amylovorum*, *Treponema medium*, *Slackia exigua* and *Treponema vincentii* (ORadj = 20.7, 7.97, 6.37 and 5.37 respectively) and the increase in *Treponema amylovorum* was statistically significant (*p* = 0.05).

**Conclusion:**

Smoking affects the subgingival bacterial profile in healthy individuals and is responsible for the depletion of beneficial bacteria and the increase in periodontopathogenic bacteria. In the CP patient group, our study suggests that subgingival bacteria (particularly *Treponema* species) make a more substantial contribution in the etiology of CP among non-smokers. Further studies using a larger sample set and more sensitive and quantitative techniques (such as real -time PCR) are needed to enhance our understanding of the exact effect of smoking on subgingival biofilm.

**Electronic supplementary material:**

The online version of this article (doi:10.1186/s12903-017-0359-4) contains supplementary material, which is available to authorized users.

## Background

Periodontitis is a group of inflammatory diseases affecting the supporting tissues of the teeth. Periodontal diseases are classified as complex disorders where environmental, lifestyle and genetic factors contribute to their etiology [1]. The most important environmental factor is the diverse microbiota arranged in a biofilm [2] which acts as an initiator of periodontal destruction, while lifestyle factors, such as smoking and oral hygiene habits, act as modifiers of disease presentation [3]. Cigarette smoking has been shown to be responsible for accelerated periodontal destruction and increased risk of periodontitis in young adults [4]. Based on epidemiological studies, smokers have more severe chronic periodontitis (CP) than non-smokers [5] and the relationship is dose-dependent [6], both in terms of number of cigarettes smoked and number of years smoking [7]. It has been suggested that increased severity of periodontitis among smokers compared to non-smokers is, in part, induced by differences in the subgingival bacterial profile [8].

Reports on the effect of smoking on the composition of the subgingival microbiota are inconclusive; some studies report a minor effect [9] and some report no effect of smoking on the subgingival microbiota [10]. However, most studies report a significant difference in the subgingival microbiota between smokers and non-smokers [8, 11–14]. These studies were all conducted on patients with periodontal disease. In contrast, studies on healthy individuals are limited with only one study investigating the effect of smoking on the subgingival microbiota of healthy young adults [15]. In order to determine the effect of smoking on subgingival bacteria, other confounding factors that would dilute or interfere with the effect of smoking, particularly those associated with periodontal disease, should be eliminated.

Therefore, the aim of the present study was to determine whether smoking affects the subgingival bacteria in healthy individuals. It also aimed to investigate if there is a difference in the subgingival microbiota between smoking induced periodontitis and periodontitis induced by factors other than smoking.

## Methods

### Study groups

The study was independently reviewed and approved by the Institutional Research Board (IRB) of Jordan University of Science and Technology (JUST). The purpose and methodology of the study, including possible future publication of clinical datasets, were fully explained to all participants or their parents if they were under the age of 18 years and their written consent was obtained before interviews, examinations and sample collection, complying with the tenets of the revised Declaration of Helsinki.

In total, 150 participants were enrolled in the study at the initial screening unit in the Dental Teaching Centre (DTC) of Jordan University of Science & Technology (JUST). Demographic data and medical history were collected, and brief extraoral and intraoral clinical examinations were performed. Patients with clinically healthy gingiva were referred to the oral diagnosis clinic and those with suspected periodontal disease were referred to the periodontics clinic, both located in the DTC. Selection criteria were: systemically healthy; Jordanian; no periodontal treatment or antimicrobial therapy for at least 6 months before the examination; at least seven natural teeth/quadrant with no fixed or removable prosthesis. Any participants using long-term medications, receiving orthodontic treatment, under the age of 16, or pregnant were excluded. Once in the designated clinic, supragingival plaque was scored and, when present, it was carefully removed. Teeth were isolated using cotton rolls, and subgingival plaque was collected prior to any periodontal examination by inserting a paper point for 30 s into the mesial sulcus of each posterior tooth apart from the third molar if present (16 sites in total). The clinical diagnosis was based on the 1999 Consensus Classification of Periodontal Diseases [16]. Four clinical variables were recorded for all participants: the periodontal pocket depth (PPD), clinical attachment loss (CAL), plaque index (PI) and gingival index (GI) [17]. The absence of periodontal disease was defined as the absence of sites with PPD of ≥4 mm in any tooth and confirmed by radiographic examination. Paper points contaminated with blood were not included as this might affect DNA quality, only points from non-bleeding sites were pooled together, placed in Eppendorf tubes and stored at –20 °C for extraction of genomic DNA and analysis. Only participants who satisfied the inclusion criteria and had a clear-cut diagnosis were included in the study and DNA was extracted from plaque samples. In total a study population of 94 unrelated individuals: 30 non-smoker CP cases (18–50 years), 9 current smoker CP cases (28–48 years), 37 non-smoker periodontally healthy subjects (17–53 years), and 18 current smoker periodontally healthy subjects (17–43 years).

### Molecular technique

Samples were placed in 2 ml phosphate-buffered saline (PBS) and incubated for 3 h at room temperature. Each sample was centrifuged at 5000 × *g* for 10 min and the supernatants removed. Genomic DNA was then extracted from the pellets using QIAamp DNA Mini Kit (Qiagen GmbH, Hilden, Germany) according to the manufacturer’s instructions.

In the first amplification, the 16S rRNA genes were amplified by PCR with universal primers 27 F and 1492R [18]. The primer sequences were: 27 F, 5′-AGA GTT TGA TCC TGG CTC AG-3′; and 1492R, 5′-TAC GGG TAC CTT GTT ACG ACT T-3′. PCR was performed in 25 μl volumes using 12.5 μl of PCR master mix (Promega, USA), 6.5 μl of nuclease free water, 1 μl of forward and reverse primers and 5 μl of DNA. Amplifications were performed using a PCR Thermal Cycler (Bio-RAD, USA) programed for 10 min at 95 °C for initial heat activation, 35 cycles of 45 s at 95 °C for denaturation, 45 s at 60 °C for annealing, 45 s at 72 °C for extension, and 7 min at 72 °C for final extension. The size of the PCR product using the universal primers was 1505 bp.

The presence or absence of the bacteria was determined by the presence or absence of the PCR band resulting from using previously reported species-specific primers [19] on the first universal PCR product as a template after being checked on Primer-BLAST (www.ncbi.nlm.nih.gov/tools/primer-blast/). Primer sequences can be found in additional word document file (see Additional file 1). PCR mixtures were prepared using 5 μl of the first PCR amplification mixture and 20 μl of the reaction mixture containing 0.5 μl of species-specific primer, 13 μl of PCR master mix (Promega, USA) and 6.5 μl of nuclease free water. PCR amplification was performed in a PCR Thermal Cycler (Bio-RAD, USA) programed for 10 min at 95 °C for initial heat activation, 35 cycles of 1 min at 95 °C for denaturation, 45 s at 55 °C for annealing, 45 s at 72 °C for extension, and 7 min at 72 °C for final extension.

The PCR products were separated on 2% agarose gels stained with ethidium bromide and visualized under a UV light transilluminator. With each gel run, negative and positive control samples were run simultaneously. In any case of uncertainty about the presence or absence of a bacteria-specific band, the PCR was repeated.

### Statistical analysis

Continuous variables were presented using mean ± SD and compared using the independent sample *t*-test, categorical variables were presented using frequencies and percentages, and were compared using Chi squared analysis. Binary logistic regression analysis was used to calculate the odds of having each bacterium in smokers compared to non-smokers for both healthy and CP patients. Smoking was the dependent variable and independent variables (covariates) were: age, gender, education level, PPD and type of bacteria. Gender, education level and type of bacteria were set as categorical variables, while age and PPD were set as continuous variables (default). Both crude and OR corrected for age, gender, education level and PPD (OR_adj_) were presented. All statistical analyses were conducted using IBM SPSS 24 (SPSS Inc., Chicago, USA). The adjusted OR (OR_adj_) was adopted for interpreting the results and a *p*-value ≤ 0.05 was considered to be statistically significant. SPSS could not calculate the OR for the bacteria if the prevalence was 100% or 0% in any of the compared groups; in that case, only the *p*-value for *χ* ^2^ analysis was shown. A power calculation was conducted to determine the statistical power of our samples to detect an association using the G*Power program [20]. The input parameters were: effect size = 0.3 (small size = 0.1, medium size = 0.3), statistical significance level α = 0.05 and two tailed.

## Results

The sociodemographic and clinical data for the participants are summarized in Table 1.

**Table 1:** Demographic, socioeconomic and clinical data for the study groups

	Healthy		Chronic periodontitis	
	Non-smoker	Smoker	Non-smoker	Smoker
	*N* = 37	*N* = 18	*N* = 30	*N* = 9
*Gender*				
Male (%)	14 (37.8)	16 (88.9)	5 (16.7)	7 (77.8)
Female (%)	23 (62.2)	2 (11.1)	25 (83.3)	2 (22.2)
*P* value	**<0.0001**		**<0.0001**	
Age (mean ± SD), years	31.84 ± 10.10	26.94 ± 8.39	38.96 ± 10.65	40.63 ± 6.72
*P* value	0.065		0.600	
*Education level*				
≥ University (%)	15 (40.5)	10 (55.6)	13 (46.4)	7 (87.5)
< University (%)	22 (59.5)	8 (44.4)	15 (53.6)	1 (12.5)
*P* value	0.294		**0.039**	
*Family income*				
Low income (%)	17 (45.9)	10 (55.6)	19 (65.5)	5 (55.6)
Middle income (%)	12 (32.4)	4 (22.2)	10 (34.5)	4 (44.4)
High income (%)	7 (18.9)	4 (22.2)	0 (0.0)	(0.0)
*P* value	0.750		0.699	
PPD (mean ± SD), mm	2.41 ± 0.82	1.87 ± 0.64	5.14 ± 0.74	4.75 ± 0.89
*P* value	**0.017**		0.284	
CAL (mean ± SD), mm	0.0 ± 0.0	0.0 ± 0.0	5.14 ± 1.06	4.88 ± 0.84
*P* value	1.000		0.471	
PI (mean ± SD)	1.41 ± 0.50	1.60 ± 0.51	2.31 ± 0.54	2.38 ± 0.74
*P* value	0.240		0.823	
GI (mean ± SD)	1.47 ± 0.62	1.60 ± 0.63	2.31 ± 0.54	2.25 ± 0.46
*P* value	0.511		0.758	
*Smoking quantitiy*				
< 10 cig/day (%)	–	3 (16.7)	–	0 (0.0)
10–20 cig/day (%)	–	11 (61.1)	–	7 (77.8)
> 20 cig/day (%)	–	4 (22.2)	–	2 (22.2)
*Smoking duration*				
< 5 years (%)	–	4 (22.2)	–	2 (22.2)
5–10 years (%)	–	10 (65.6)	–	4 (44.5)
> 10 years (%)	–	4 (22.2)	–	3 (33.3)

Significant *P* values ≤ 0.05 are shown in bold

In our sample set, a significant difference in gender was observed between smokers and non-smokers in the two groups, where males dominated the smokers groups (*p* < 0.0001). The PPD was significantly higher among the healthy non-smokers group compared to healthy smokers. No significant difference was observed in the oral hygiene habits between the four investigated groups (data not shown).

**Fig. 1:**
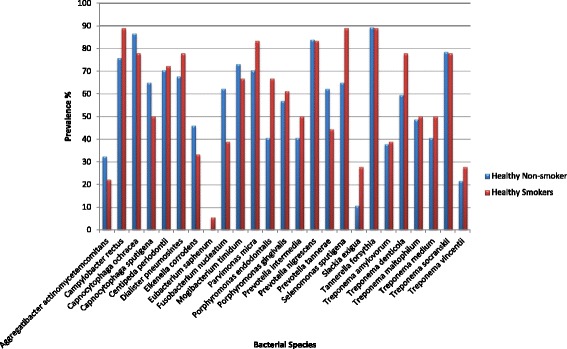
Prevalence of bacterial species in healthy smokers and healthy non-smokers

The frequency of bacteria and the odds of having each of the 25 bacteria among healthy smokers compared to healthy non-smokers are presented in Fig. 1 and Table 2, respectively. Both crude and adjusted regression analyses are presented in Table 2. The odds of having 15 bacteria was increased in healthy smokers compared to healthy non-smokers, and the highest increases were detected in *Slackia exigua, Selenomonas sputigena*, and *Campylobacter rectus* (OR_adj_ =10.1, 6.62 and 5.62, respectively). The odds of having eight bacteria was decreased among healthy smokers, and the decrease was mostly noted in *Prevotella nigrescens*, *Capnocytophaga ochracea,* and *Eikenella corrodens* (OR_adj_ = 0.31, 0.32 and 0.35, respectively). None of these differences were statistically significant. *Eubacterium saphenum* was not present among non-smokers.

**Table 2:** Odds of having each bacterium detected in plaque samples of healthy smokers compared to healthy non-smokers

Bacterial species	Crude OR (95% CI)	P	Adjusted^a^ OR (95% CI)	*P*-value
*Aggregatibacter actinomycetemcomitans*	0.57 (0.2–2.1)	0.40	0.76 (0.1–4.1)	0.74
*Campylobacter rectus*	2.67 (0.5–13.9)	0.25	5.62 (0.4–73.3)	0.19
*Capnocytophaga ochracea*	0.57 (0.1–2.4)	0.44	0.32 (0.1–2.4)	0.26
*Capnocytophaga sputigena*	0.57 (0.2–1.8)	0.33	0.41 (0.1–2.3)	0.31
*Centipeda periodontii*	1.14 (0.3–4.0)	0.83	0.60 (0.1–3.8)	0.58
*Dialister pneumosintes*	1.75 (0.5–6.5)	0.40	2.10 (0.3–14.0)	0.44
*Eikenella corrodens*	0.56 (0.2–1.8)	0.33	0.35 (0.1–1.8)	0.21
*Eubacterium saphenum*	–	–	–	–
*Fusobacterium nucleatum*	0.36 (0.1–1.2)	0.09	0.57 (0.1–2.7)	0.48
*Mogibacterium timidum*	0.67 (0.2–2.3)	0.52	0.41 (0.1–3.0)	0.38
*Parvimonas micra*	2.20 (0.6–9.2)	0.28	3.22 (0.2–48.8)	0.40
*Porphyromonas endodontalis*	0.25 (0.0–2.3)	0.22	1.54 (0.3–7.3)	0.58
*Porphyromonas gingivalis*	1.26 (0.4–4.0)	0.70	4.37 (0.7–26.5)	0.11
*Prevotella intermedia*	1.40 (0.5–4.4)	0.56	3.95 (0.6–24.1)	0.14
*Prevotella nigrescens*	0.14 (0.0–1.1)	0.06	0.31 (0.0–4.7)	0.40
*Prevotella tannerae*	0.51 (0.2–1.6)	0.25	0.41 (0.1–2.1)	0.28
*Selenomonas sputigena*	4.52 (0.9–22.8)	0.07	6.62 (0.9–50.5)	0.07
*Slackia exigua*	3.08 (0.7–13.3)	0.13	10.1 (0.7–146.1)	0.09
*Tannerella forsythia*	1.00 (0.2–6.1)	1.00	2.68 (0.2–29.9)	0.42
*Treponema amylovorum*	6.05 (1.1–34.4)	0.04	4.38 (0.5–33.1)	0.15
*Treponema denticola*	2.50 (0.7–9.1)	0.17	1.87 (0.3–12.4)	0.51
*Treponema maltophilum*	1.12 (0.4–3.5)	0.85	1.26 (0.2–6.8)	0.79
*Treponema medium*	1.57 (0.5–4.9)	0.44	1.1 (0.2–5.6)	0.91
*Treponema socranskii*	1.00 (0.3–3.9)	1.00	0.92 (0.1–6.7)	0.93
*Treponema vincentii*	8.29 (0.7–104.9)	0.10	1.41 (0.2–9.2)	0.72

^a^Adjusted for gender and age

**Fig. 2:**
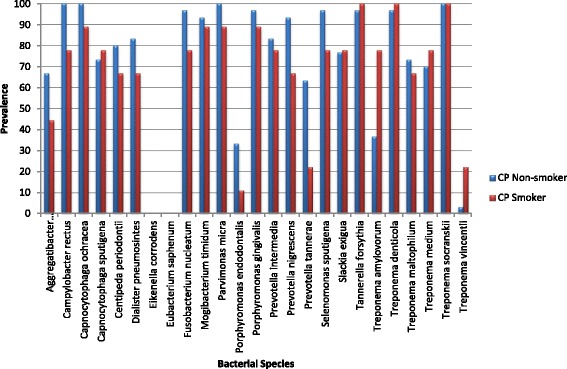
Prevalence of bacterial species in CP patients who were smokers or non-smokers

Within the CP group, when smokers were compared to non-smokers, the prevalence of six bacteria was increased among smokers (Fig. 2). When correcting for confounders, *Treponema amylovorum, Treponema medium, Slackia exigua* and *Treponema vincentii* had the highest odds ratios (OR_adj_ = 20.7, 7.97, 6.37 and 5.37, respectively) and the increase in *Treponema amylovorum* was statistically significant (*p* = 0.05) (Table 3). *Treponema denticola*, *Treponema socranskii* and *Tannerella forsythia* were present in all CP smokers. The remaining bacteria were decreased among CP smokers (Table 3).

**Table 3:** Odds of having each bacterium detected in plaque samples of CP smokers compared to CP non-smokers

*Bacterial species*	Crude OR (95% CI)	*P*-value	Adjusted^a^ OR (95% CI)	*P*-value
*Aggregatibacter actinomycetemcomitans*	0.40 (0.1–1.8)	0.24	0.53 (0.07–4.4)	0.57
*Campylobacter rectus*	–		–	0.01*
*Capnocytophaga ochracea*	–		–	0.06*
*Capnocytophaga sputigena*	1.27 (0.2–7.5)	0.79	4.8 (0.2–108.1)	0.32
*Centipeda periodontii*	0.50 (0.1–2.6)	0.41	0.88 (0.1–8.4)	0.91
*Dialister pneumosintes*	0.40 (0.1–2.2)	0.29	0.76 (0.07–8.2)	0.82
*Eikenella corrodens*	–		–	–
*Eubacterium saphenum*	–		–	–
*Fusobacterium nucleatum*	0.12 (0.0–1.5)	0.10	0.52 (0.0–44.3)	0.77
*Mogibacterium timidum*	0.57 (0.1–7.1)	0.66	0.09 (0.0–2.0)	0.13
*Parvimonas micra*	–		–	0.06*
*Porphyromonas endodontalis*	0.25 (0.0–2.3)	0.22	0.25 (0.02–3.5)	0.31
*Porphyromonas gingivalis*	0.28 (0.0–4.9)	0.38	0.19 (0.01–5.3)	0.32
*Prevotella intermedia*	0.70 (0.1–4.4)	0.70	0.78 (0.05–13.3)	0.87
*Prevotella nigrescens*	0.14 (0.0–1.0)	0.06	0.66 (0.04–10.3)	0.77
*Prevotella tannerae*	0.17 (0.0–0.9)	0.04	0.57 (0.1–5.7)	0.63
*Selenomonas sputigena*	0.12 (0.0–1.5)	0.10	0.34 (0.0–46.5)	0.66
*Slackia exigua*	1.07 (0.2–6.4)	0.95	6.37 (0.3–146.1)	0.25
*Tannerella forsythia*	–		–	*0.58
*Treponema amylovorum*	6.05 (1.1–34.4)	0.04	20.7 (0.9–466.0)	**0.05**
*Treponema denticola*	–		–	*0.58
*Treponema maltophilum*	0.73 (0.2–3.6)	0.70	0.79 (0.1–7.2)	0.84
*Treponema medium*	1.50 (0.3–8.7)	0.65	7.97 (0.4–67.1)	0.23
*Treponema socranskii*	–		–	1.00*
*Treponema vincentii*	8.29 (0.7–104.9)	0.001	5.37 (0.2–151.8)	0.32

*P value based on *χ* ^2^ analysis

^a^Adjusted for gender and education level

Significant *P* values ≤ 0.05 are shown in **bold**

Power calculations revealed that the sample set had 62.9 and 48.1% power to detect an association for healthy and CP groups, respectively, when the significance level was set at 0.05.

## Discussion

This study reports the effect of smoking on the bacterial profile of healthy and CP subjects by comparing the bacterial profile of 25 bacterial species among smokers and non-smokers. These particular bacterial species were analyzed because there is previous evidence of their association with periodontal diseases [19, 21]. Among healthy individuals, the bacteria present among smokers differ from those present among non-smokers although both are periodontitis-free groups (CAL = 0.00). A moderate increase in the red complex bacteria was observed among smokers compared to non-smokers, which agrees with previously reported results [15]. However, smoking was responsible for increasing the prevalence of other periodontopathogenic bacteria in subjects with healthy gingiva that were also prevalent in CP patients. After ruling out the effects of other confounding factors, smoking was responsible for increasing the prevalence of *Slackia exigua, Selenomonas sputigena*, and *Campylobacter rectus* by 910%, 562%, and 462% times, respectively. Furthermore, smoking was responsible for decreasing the prevalence of bacteria that were considered to be less periodontopathogenic, for instance, *Eikenella corrodens* was 65% less likely to be present among healthy smokers. These findings indicate that smoking is responsible for the depletion of beneficial bacteria and increasing the abundance of periodontopathogenic bacteria among healthy individuals.

When comparing the bacterial profiles of smoker and non-smoker CP patients, an unexpected observation was the increased prevalence and the odds of having many of the investigated bacterial species among the non-smokers. Exceptions were limited to *Treponema* species and *Slackia exigua* which were increased among CP smokers. Previous reports concluded that smoking is responsible for enriching the subgingival microbiota with pathogenic species [14, 22]. This effect was supported by more recent studies conducted on CP patients, which concluded that smoking has a detrimental impact on the periodontal status and the microbial profile [11, 23]. This was further supported by a longitudinal study investigating the effect on bacteria of smoking cessation after 6 and 12 months among CP patients [24]. Our results support the view that smoking affects the subgingival microbiota in CP patients and that it is responsible for the increase in the *Treponema* species and *Slackia exigua*. They also suggest that, in non-smoker CP patients, bacteria tend to play a more crucial role in pathogenesis, where a larger number of bacterial species will be involved in constructing the subgingival plaque, while in smoker CP patients, smoking is more likely to contribute to the disease by inducing changes within gingival tissues and the gingival sulcus. Smoking has been reported to affect the immune response against bacteria [25] by lowering gingival crevicular fluid (GCF) volume [26, 27] or by affecting the host antibody response [28], through reducing sera immunoglobulin G antibody titers against the subgingival bacteria [10].

When looking at the whole sample set, smoking had two distinct effects on subgingival bacteria: some bacteria were more prevalent among smokers as observed with *Tannerella forsythia*, *Treponema denticola* and *Treponema medium*, while other bacteria were less prevalent among smokers as observed with *Aggregatibacter actinomycetemcomitans*, *Fusobacterium nucleatum*, *Prevotella tannerae*, *Capnocytophaga ochracea* and *Mogibacterium timidum.* Based on these observations, smoking might induce changes in the oral and gingival ecosystem that make the environment more favorable for some bacterial species to grow, while for others, these changes make the environment less favorable for growth.

Gender was found to be significantly different between smokers and non-smokers of both groups. This was mainly attributed to the fact that the prevalence of smoking is much higher in males compared to females in the Jordanian population [29, 30]. The prevalence of smoking in Jordanian men is reported to be in the range of 56.9–62% compared to 11.0–11.4% in women [29, 30]. The effect of gender on the subgingival bacterial flora is controversial; several studies conducted on periodontitis patients have reported a difference in the bacterial profile between males and females [31, 32], while other studies have reported that gender has no effect on the subgingival bacterial profile in CP patients or in healthy controls [33, 34]. The PPD was significantly higher in healthy non-smokers compared to smokers; this increase is most likely related to increased age among the non-smokers.

This study investigated the effect of smoking on the prevalence of bacterial species; however, no changes in the serotype induced by smoking could be determined. Furthermore, no quantitative judgments on the amount of bacteria could be made as this requires using a real-time PCR technique, which was not available to us. An effect of smoking on the immune response [10, 25–28] could not be ruled out, and this would provide another mechanism of action in inducing periodontal disease. Finally, although differences in the bacterial profile could be detected in this sample set, only the increase in *Treponema amylovorum* in CP smokers was statistically significant (*p* = 0.05); other differences were not statistically significant (p > 0.05) due to limited power related to the small sample number, which is one of the limitations of the study; hence, conclusions based on these results should be interpreted with caution.

## Conclusions

Smoking affects the subgingival bacterial profile in healthy individuals and is responsible for the depletion of beneficial bacteria and the increase in periodontopathogenic bacteria. In the CP patient group, our study suggests that subgingival bacteria (particularly *Treponema* species) might make a more substantial contribution in the etiology of CP among non-smokers. Further studies using a larger sample set and more sensitive and quantitative techniques (such as real-time PCR) are needed to enhance our understanding of the exact effect of smoking on subgingival biofilm.

## Additional file

Additional file 1: Target Bacteria and Their Species-Specific Primers. A table including names of bacteria investigated and the species-specific primers (forward and reverse) used for the PCR. (DOCX 16 kb)

## Abbreviations

bp: Base pair
CAL: Clinical attachment loss
CP: Chronic periodontitis
DTC: Dental teaching centre
F: Forward
g: Relative centrifugal force
GCF: Gingival crevicular fluid
GI: Gingival index
IRB: Institutional Research Board
JUST: Jordan University of Science and Technology
min: Minute
OR: Odds ratio
OR_adj_: Adjusted odds ratio
PBS: Phosphate-buffered saline
PCR: Polymerase chain reaction
PI: Plaque index
PPD: Periodontal pocket depth
R: Reverse
rRNA: Ribosomal ribonucleic acid
s: Seconds
SD: Standard deviation.
