# Supplementary material for: Effect of cigarette smoking on subgingival bacteria in healthy subjects and patients with chronic periodontitis
Source: BMC Oral Health. 2017 Mar 21;17:64. doi: 10.1186/s12903-017-0359-4 (PMC5361727; doi:10.1186/s12903-017-0359-4)
Supplement: Additional file 1: — Target Bacteria and Their Species-Specific Primers. A table including names of bacteria investigated and the species-specific primers (forward and reverse) used for the PCR. (DOCX 16 kb) [file 12903_2017_359_MOESM1_ESM.docx]

**Target Bacteria and Their Species-Specific Primers**

| **Species** | **Sequence (5`-3`)** | **Size (bp)** |
| --- | --- | --- |
| *Aggregatibacter actinomycetemcomitans* | F: CTCAGAGATGGGTTTGTGCC  R: AGATTCACTCCCCATCGCTG | 273 |
| Campylobacter rectus | F: TTTCGGAGCGTAAACTCCTTTTC  R: TTTCTGCAAGCAGACACTCTT | 598 |
| Capnocytophaga ochracea | F: AGAGTTTGATCCTGGCTCAG  R: GATGCCGTCCCTATATACTATGGGG | 185 |
| Capnocytophaga sputigena | F: AGAGTTTGATCCTGGCTCAG  R: GATGCCGCTCCTATATACCATTAGG | 185 |
| Centipeda periodontii | F: AGAGTTTGATCCTGGCTCAG  R: TTACAAAGGATTATTCGCCC | 450 |
| Dialister pneumosintes | F: TTCTAAGCATCGCATGGTGC  R: GATTTCGCTTCTCTTTGTTG | 1105 |
| Eikenella corrodens | F: CGATTAGCTGTTGGGCAACTT  R: ACCCTCTGTACCGACCATTGTAT | 410 |
| Eubacterium saphenum | F: TCTACTAAGCGCGGGGTGA  R: ACCCGATTAAGGGTAC | 430 |
| Fusobacterium nucleatum | F: GAAGAAACAAATGACGGTAACAAC  R: GTCATCCCCACCTTCCTCCT | 705 |
| *Parvimonas micra* | F: TCGAACGTGATTTTTGTGGA  R: TCCAGAGTTCCCACCTCT | 1074 |
| Mogibacterium timidum | F: AAGCTTGGAAATGACGC  R: CCTTGCGCTTAGGTAA | 524 |
| Porphyromonas endodontalis | F: GCTGCAGCTCAACTGTAGTC  R: CCGCTTCATGTCACCATGTC | 672 |
| Porphyromonas gingivalis | F: GCGTATGCAACTTGCCTTAC  R: GTTTCAACGGCAGGCTGAAC | 518 |
| Prevotella intermedia | F: CGTGGACCAAAGATTCATCGGTGGA  R: CCGCTTTACTCCCCAACAAA | 259 |
| Prevotella nigrescens | F:GGTTTCATTGACGGCATCCGATATGAAACR: CACGTCTCTGTGGGCTGCGA | 828 |
| Prevotella tannerae | F: CTTAGCTTGCTAAGTATGCCG  R: AGCTGACTTATACTCCCG | 550 |
| Selenomonas sputigena | F: AGAGTTTGATCCTGGCTCAG  R: TCAATATTCTCAAGCTCGGTT | 478 |
| Slackia exigua | F: GCCAAGCGGCCTCGTCGAAG  R: CGGCTTTAAGGGATTCGCTCG | 697 |
| Tannerella forsythia | F: AAAACAGGGGTTCCGCATGG  R: CACCGCGGACTTAACAGC | 426 |
| Treponema amylovorum | F: AGAGTTTGATCCTGGCTCAG  R: CACGCCTTTATTCCGTGAG | 193 |
| Treponema denticola | F: TAATACCGAATGTGCTCATTTACAT  R: TCAAAGAAGCATTCCCTCTTCTTCTTA | 316 |
| Treponema maltophilum | F: AGAGTTTGATCCTGGCTCAG  R: CTATTGTGCTTATTCATCAGGC | 438 |
| Treponema medium | F: CACTCAGTGCTTCATAAGGG  R: CGGCCTTATCTCTAAGACC | 856 |
| Treponema socranskii | F: AGGTAGACAGCGGGAAAGGA  R: AACCCAACACCTCACGGCA | 902 |
| Treponema vincentii | F: GTCTCAATGGTTCATAAGAA  R: CAAGCCTTATCTCTAAGACT | 856 |
